# Supplementary material for: REM sleep is associated with distinct global cortical dynamics and controlled by occipital cortex
Source: Nat Commun. 2022 Nov 12;13:6896. doi: 10.1038/s41467-022-34720-9 (PMC9653484; doi:10.1038/s41467-022-34720-9)
Supplement: Supplementary file 1 — Supplementary Information [file 41467_2022_34720_MOESM1_ESM.pdf]

REM Sleep is Associated with Distinct Global Cortical Dynamics and Controlled by Occipital Cortex

Supplementary figures and legends

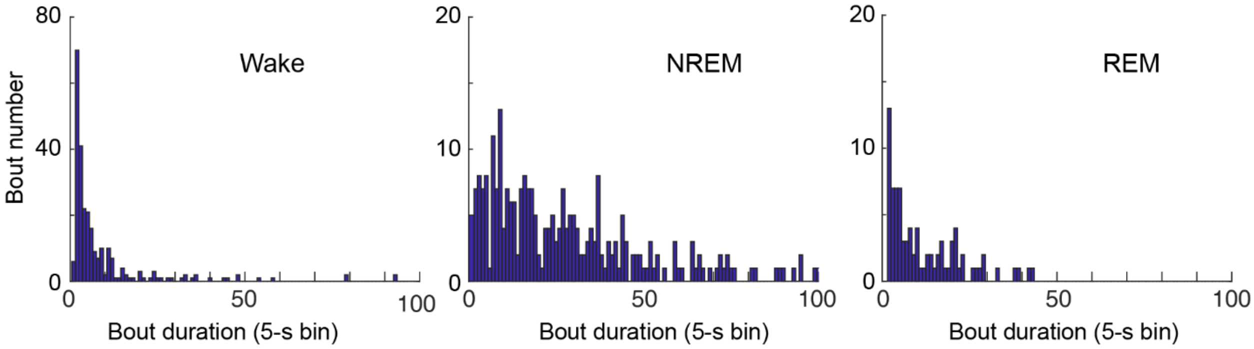

Supplementary Fig. 1 | Sleep architecture of mice during the mesoscale Ca^{2+} imaging (related to Fig. 1).

Shown is the distribution of bout duration for each sleep-wake state. *n* = 15 recordings from 5 mice.

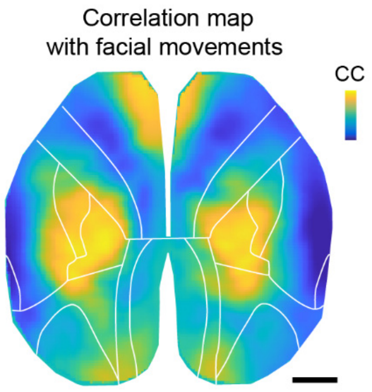

Supplementary Fig. 2 | Cortical activity associated with facial movements (related to Fig. 1).

Shown is the mean correlation map between cortical activity and facial movements. *n* = 9 recordings from 5 mice. Scale, CC -0.3 – 0.3. Black bar, 1 mm.

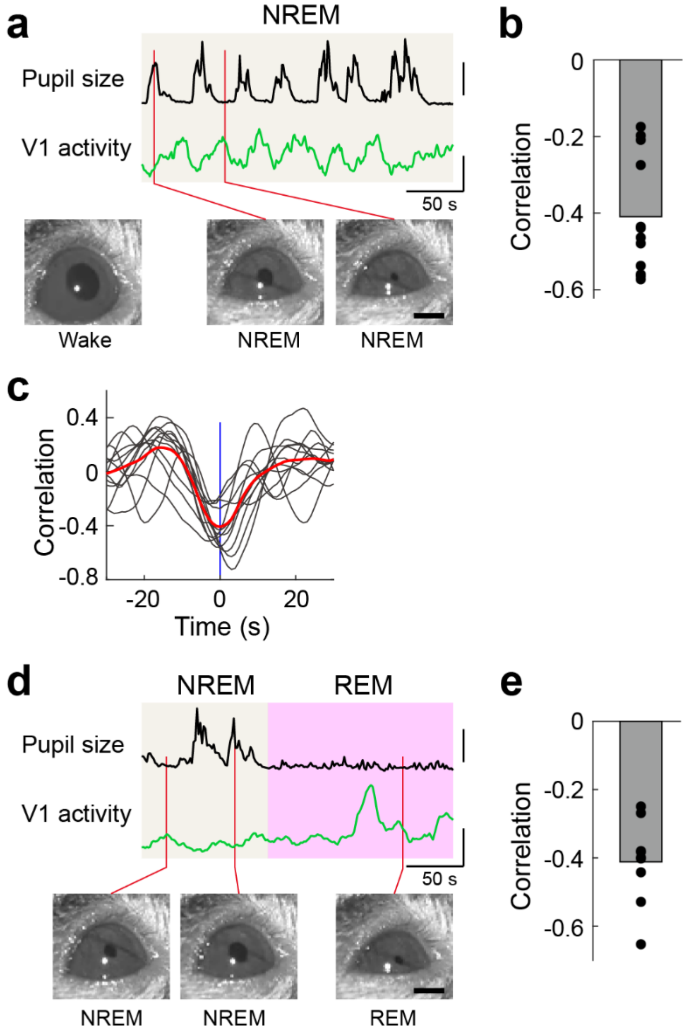

Supplementary Fig. 3 | Relationship between pupil size and V1 activity during recording (related to Fig. 1).

a An example of pupil size (Scale, 1 mm) and V1 activity (Scale, $\Delta F/F_0$, 1 z-score) during NREM sleep. **b-c** Quantification of cross-correlation between the two signals. In **b**, *n* = 12. In **c**, *n* = 12. **d-e** Same as **a-b**, except that analysis was performed for data during the NREM to REM transition period. In **d**, (Scale, 1 mm and $\Delta F/F_0$, 1 z-score). In (**e**), *n* = 9. All black bars in the figure represent 1 mm. Raw data for **b** and **e** are provided in a Source Data file.

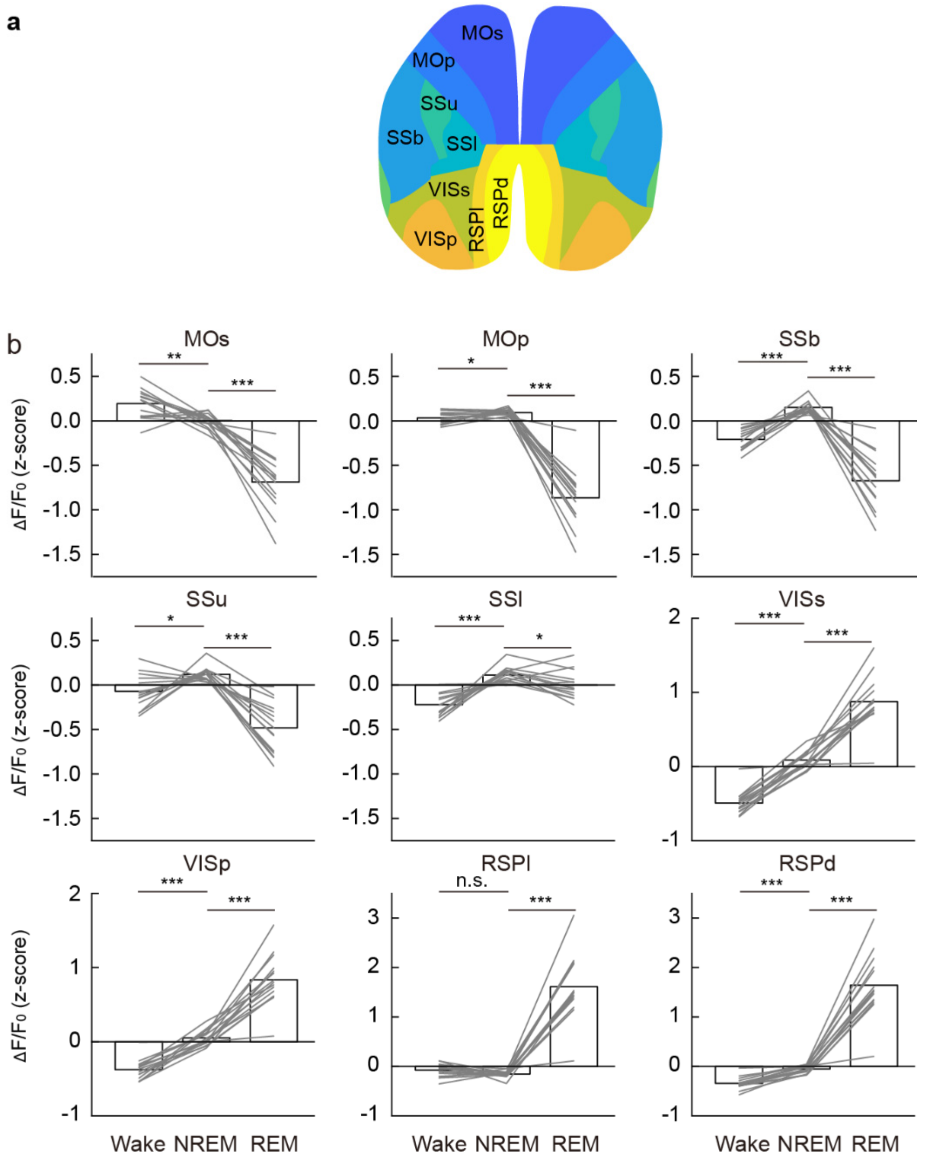

Supplementary Fig. 4 | Normalized activity in each brain region during the sleep-wake cycle (related to Fig. 1).

a Schematic diagram of the anatomical atlas used for alignment. **b** Quantification of the normalized (z-score) activity in each region during the sleep-wake cycle. The averaged Ca^{2+} activity in each region during each recording was first z-score normalized, and then mean activity in the three brain states was computed. n = 15 recording sessions from 5 mice. *$P < 0.05$, **$P < 0.01$, ***$P < 0.001$; Student's paired *t*-test or Wilcoxon signed-rank test. MOs, secondary motor cortex; MOp, primary motor cortex; SSb, somatosensory cortex, barrel field; SSu, somatosensory cortex, upper limb; SSl, somatosensory cortex, lower limb; VISp, primary visual cortex; VISs, association

visual cortex; RSPd, dorsal retrosplenial cortex; RSPl, lateral retrosplenial cortex. Raw data for **b** are provided in a Source Data file.

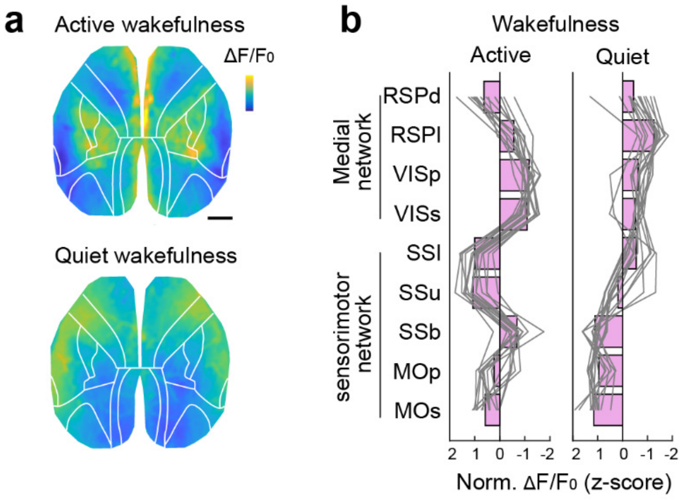

Supplementary Fig. 5 | Normalized activity in each brain region during active and quiet wakefulness (related to Fig. 1).

a Average Ca^{2+} activity during active and quiet wakefulness. Scale ($\Delta F/F_0$, z-score): -1.2 – 1.2. The black bar is 1 mm. **b** Normalized activation across the recorded brain regions in active and quiet wakefulness. *n* = 15 sessions from 5 mice. Raw data for **b** are provided in a Source Data file.

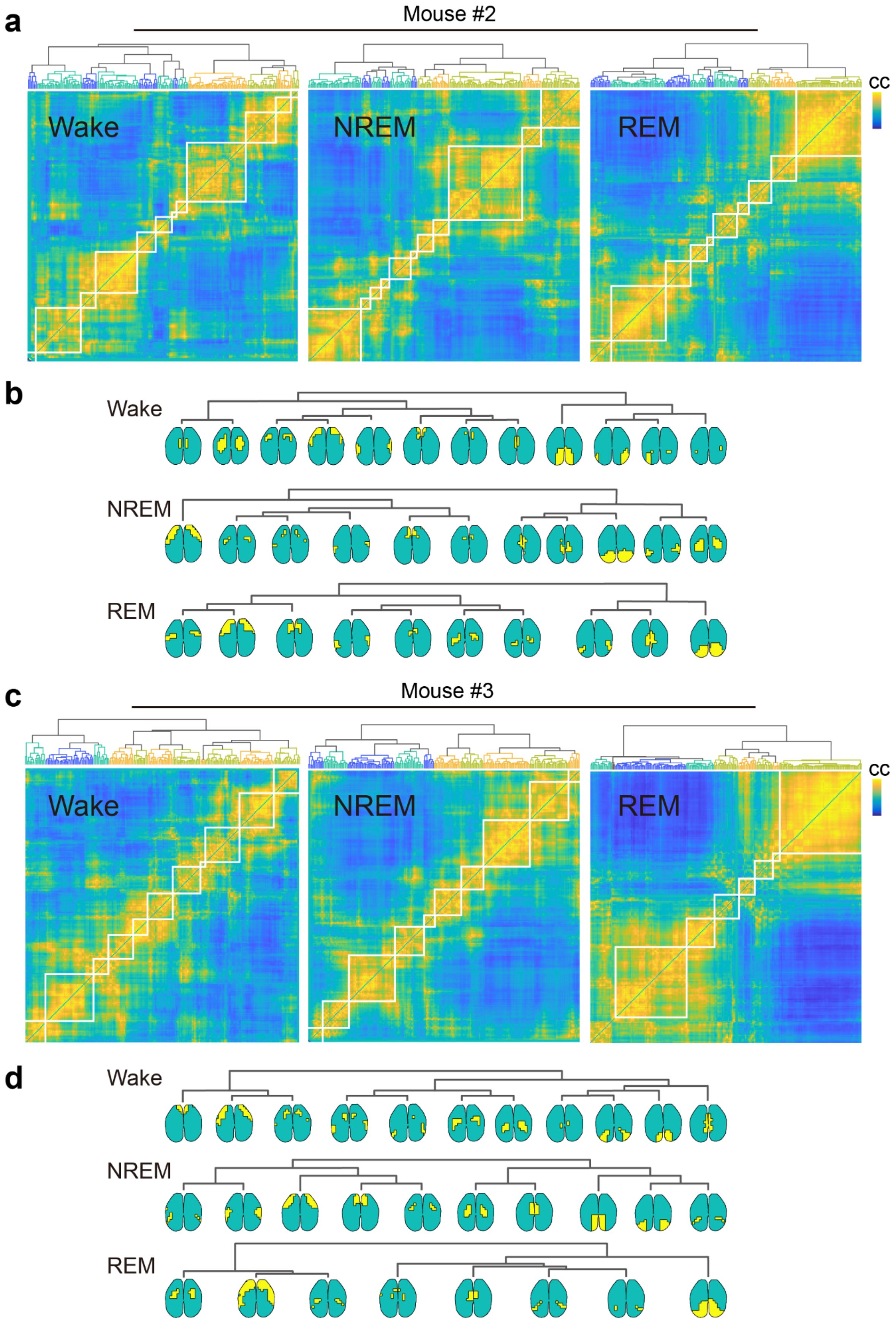

a
Mouse #2
cc
Wake
NREM
REM
b
Wake
NREM
REM
c
Mouse #3
cc
Wake
NREM
REM
d
Wake
NREM
REM

Supplementary Fig. 6 | Regional activity correlation matrix during each sleep wake state (related to Fig. 2)

a Regional activity correlation matrix during Wake (left), NREM (middle), and REM (right) in one example recording (~70 minutes). **b** Cortical networks revealed by the hierarchical clustering analysis. **c-d** Same as **a-b**, except that data were from a different mouse. All color scales are -1 – 1.

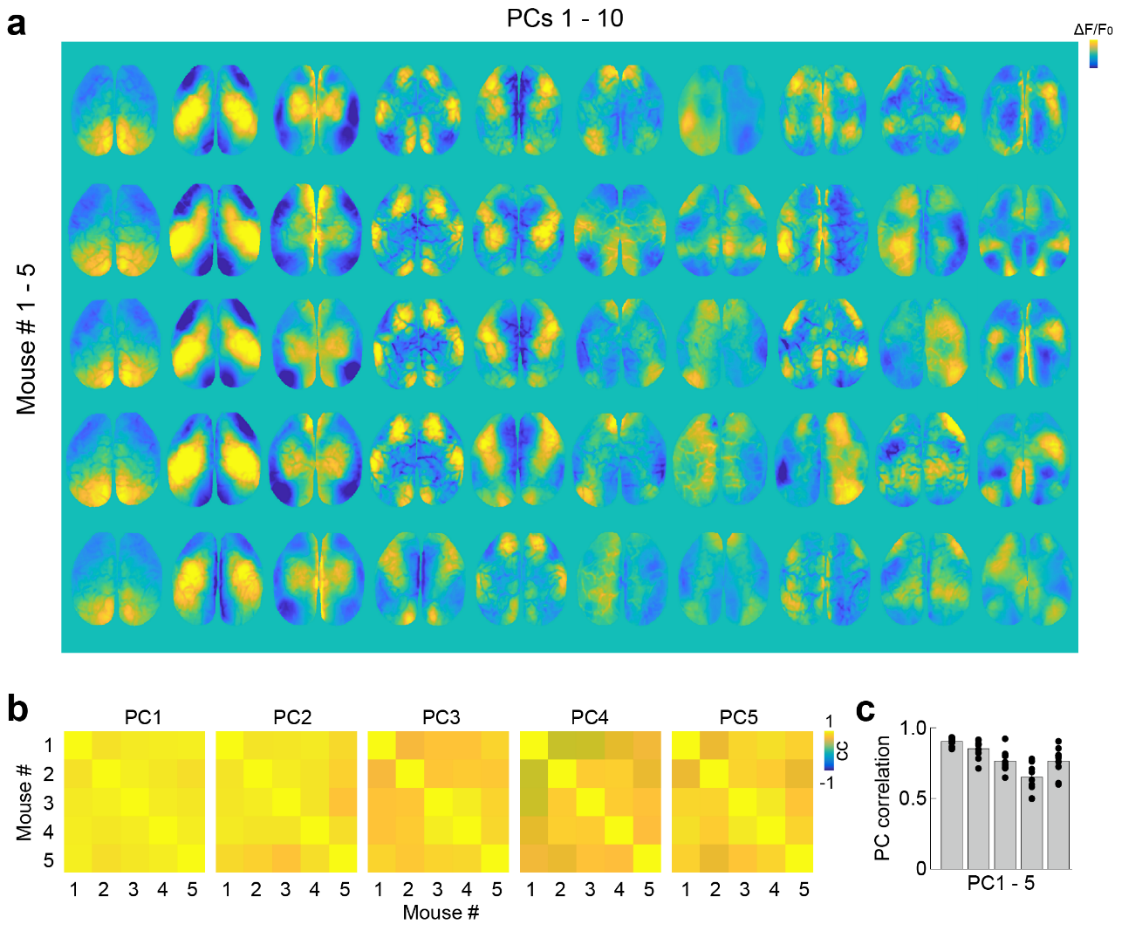

Supplementary Fig. 7 | PCA of neocortical activity patterns during the sleep-wake cycle (related to Fig. 3)

a The first ten PCs in each mouse. The first five PCs in mouse #4 (the 4th row from the top) were also shown in Fig. 3a. Scale ($\Delta F/F_0$, norm. z-score): -1 – 1. **b** Cross-correlation of PC1-5 across mice. Note that PC4 and PC5 were swapped during analysis. **c** Quantification of pairwise cross-correlation in **b**. Data were from 5 mice. Raw data for **c** are provided in a Source Data file.

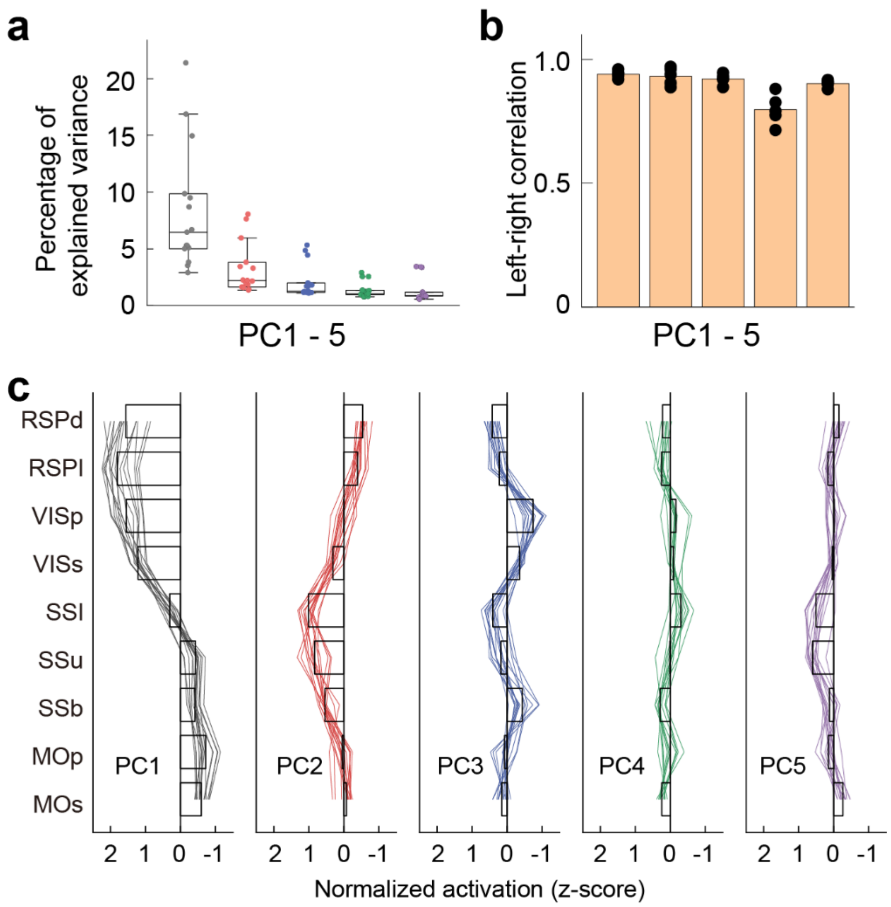

Supplementary Fig. 8 | Additional analysis for the PCA results in Figure 3 (related to Fig. 3).

a Percentage of explained variance of the five major PCs. Data of PC1 - PC5 were from 15, 15, 15, 13, and 14 sessions from 5 mice (2 or 3 sessions / mouse), respectively. The box plot shows a 25% -75% range, the whisker shows a range within the 1.5 interquartile range, and the line represents the median. **b** Cross-correlation between PC1-5 in the two hemispheres. *n* = 5 recordings from 5 mice (1 recordings / mouse). **c** Normalized activation of the five major PCs in Figure 2A in each brain region. Each line represents data from one recording. Data of PC1 - PC5 were from 15, 15, 15, 13, and 14 sessions from 5 mice (2 or 3 sessions / mouse), respectively. Raw data are provided in a Source Data file.

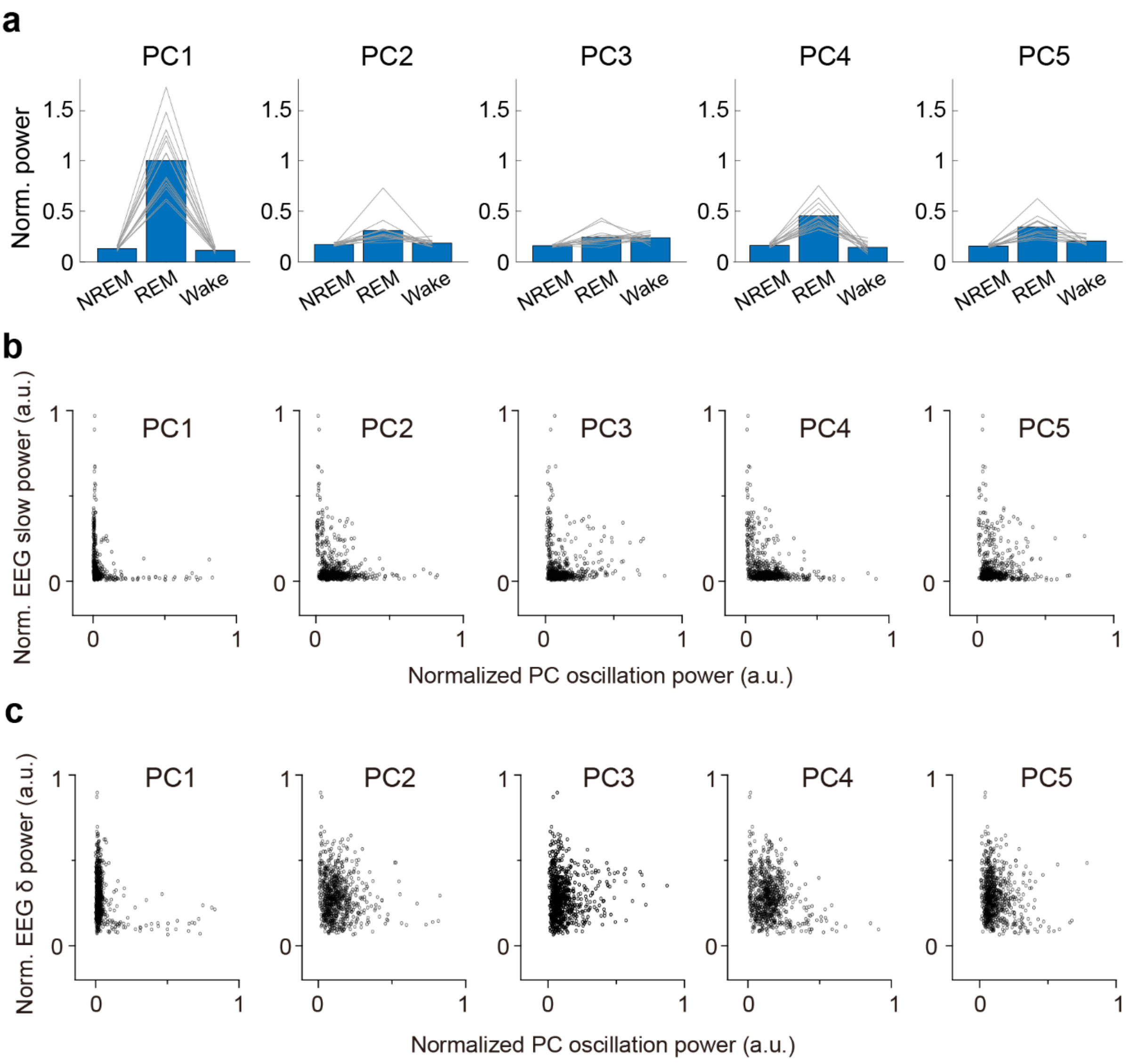

Supplementary Fig. 9 | Analysis of oscillations in each PCs (related to Fig. 3)

a Normalized power of oscillation (0 - 5 Hz) in each PCs during different brain states. n = 15 recording. **b** Lack of correlation between the oscillation power in EEG slow wave (< 1Hz) and the PCs. **c** Lack of correlation between the oscillation power in EEG δ band (0.5 – 4 Hz) and the PCs. Raw data are provided in a Source Data file.

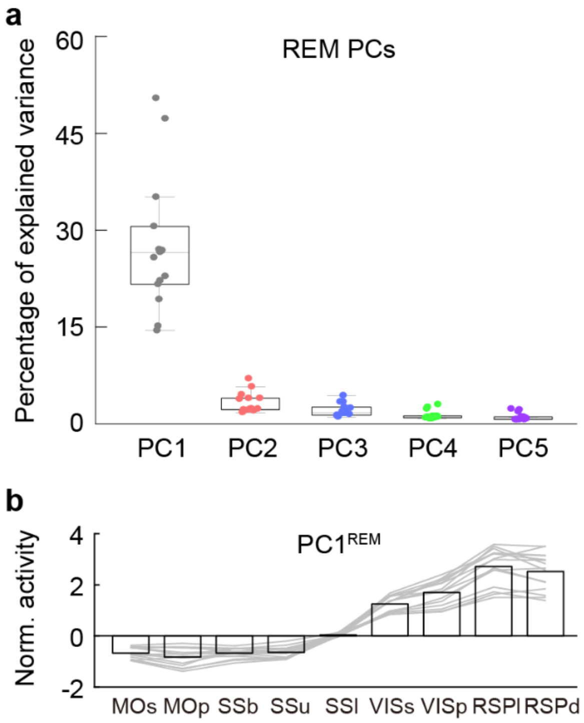

Supplementary Fig. 10 | Percentage of explained variance of PC1-5 of REM and normalized activation of PC1^{REM} in each brain region (related to Fig. 4)

a Percentage of explained variance of the five major PCs of REM activity. Data of PC1 - PC5 were from 15 sessions from 5 mice, respectively (3 sessions / mouse). The box plot shows a 25% –75% range, the whisker shows a range within the 1.5 interquartile range, and the line represents the median. **b** Normalized activation of PC1^{REM} in each brain region. Each line represents data from one recording. n = 15 sessions from 5 mice (3 sessions / mouse). Raw data are provided in a Source Data file.

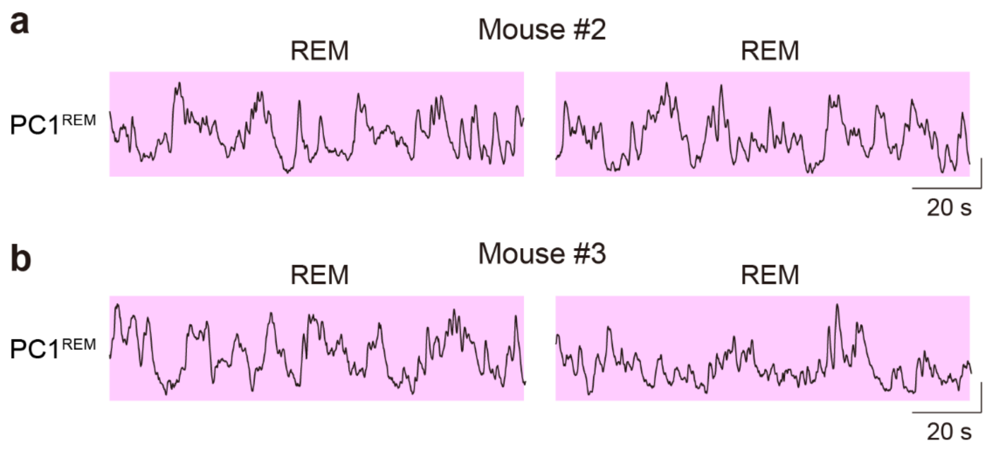

Supplementary Fig. 11 | Dynamic changes in PC1^{REM} from two other mice (related to Fig. 4)

Shown are two additional examples of PC1^{REM} from two other mice. Scale, 0.03.

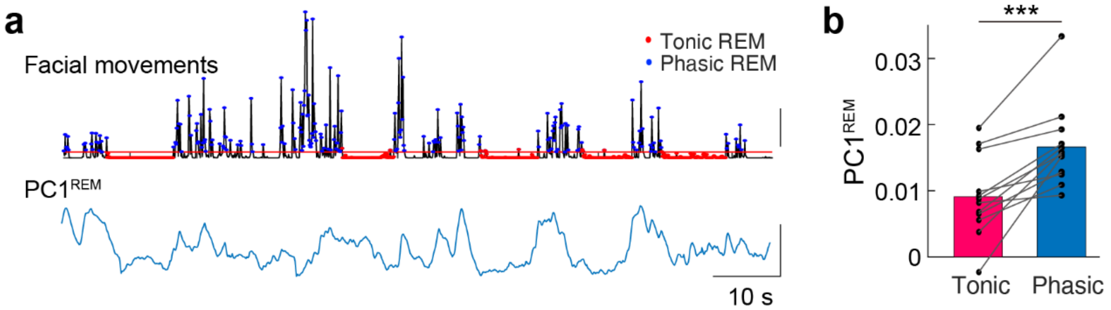

Supplementary Fig. 12 | PC1^{REM} during tonic and phasic REM (related to Fig. 4)

a An example of facial movements (scale, 200 a.u.) and PC1^{REM} (scale, 0.03). The Red line indicates the mean. **b** Quantification of PC1^{REM} in tonic and phasic REM. $P < 0.001$, Wilcoxon signed-rank test. n = 12 sessions from 5 mice. Raw data for **b** are provided in a Source Data file.

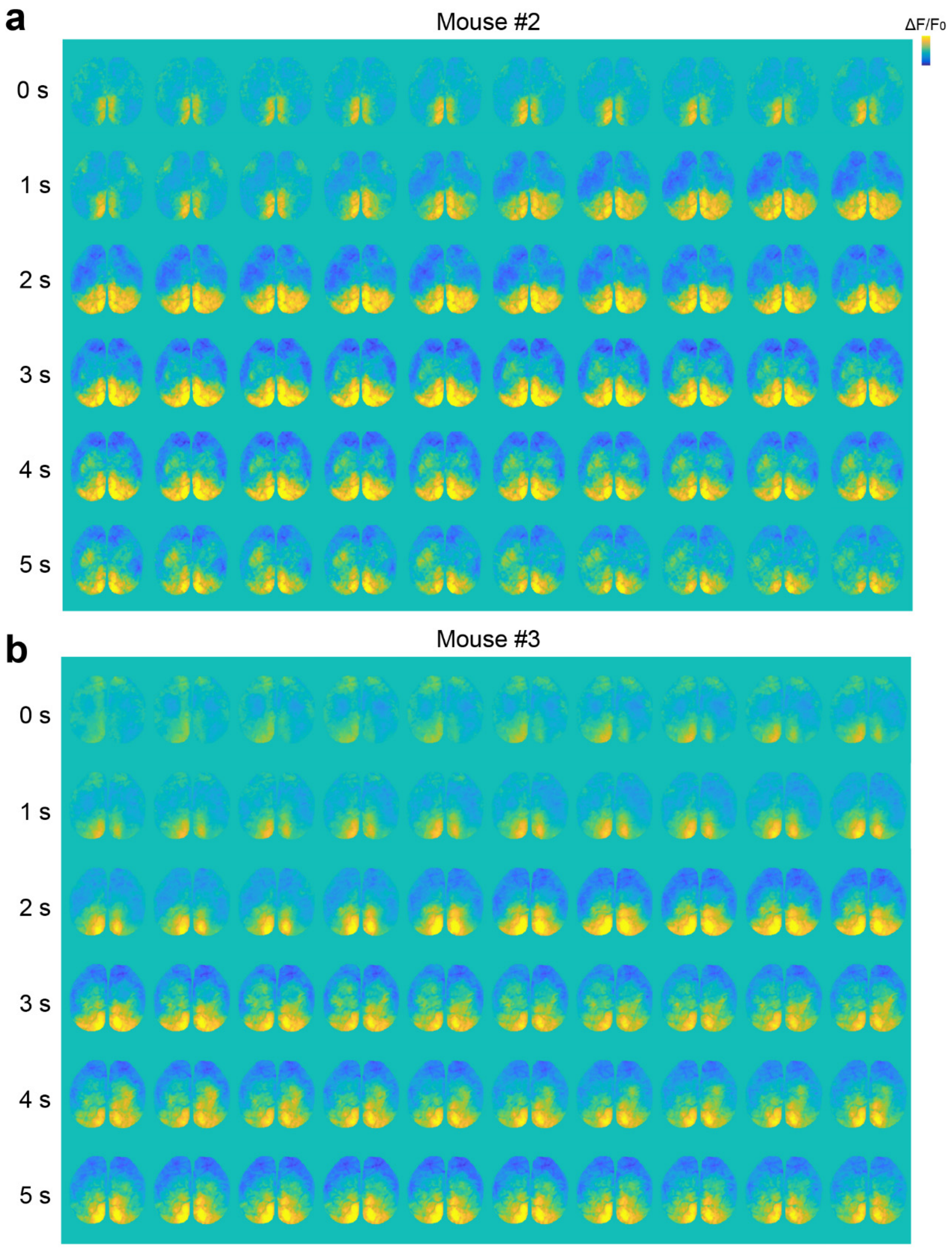

Supplementary Fig. 13 | Spreading activity during REM sleep (related to Fig. 4)

Shown are two examples of the spreading activity during REM sleep from two other mice. Images were acquired with a frame rate of 10 Hz. Scale ($\Delta F/F_0$, norm. z-score): -1 – 1.

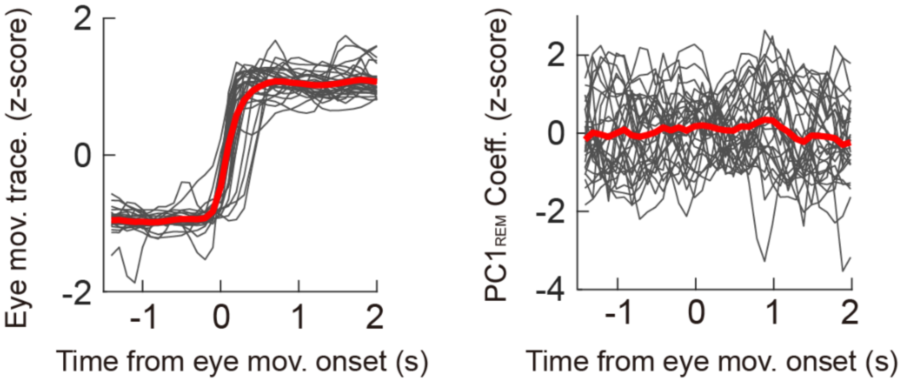

Supplementary Fig. 14 | Lack of correlation between eye movements and occipital activity during wakefulness (related to Fig. 4)

Shown are eye movements (left) and the associated occipital activity (right). n = 29 eye-movement events from 5 mice. Black trace, data from each event; Red trace, group average.

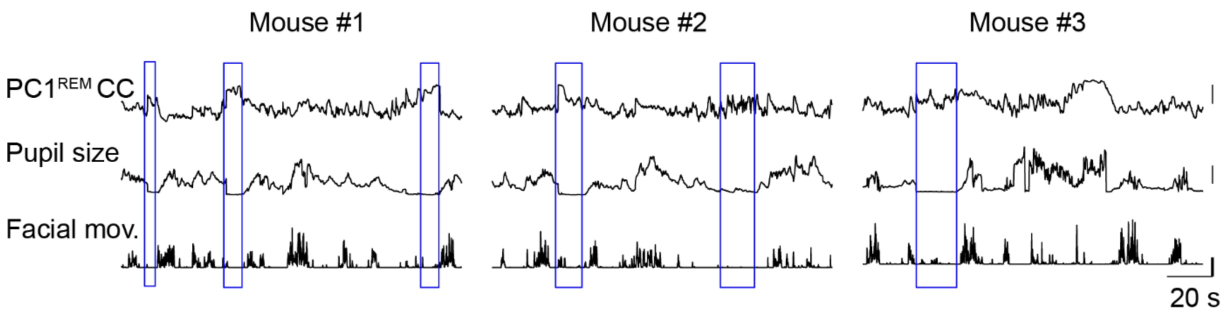

Supplementary Fig. 15 | PC1^{REM}-like activity during drowsy states. (related to Fig. 5)

Shown are three examples of PC1^{REM}-like activity during drowsy states. Drowsy states (highlighted in the blue box) were manually identified when the pupil was constricted and lack of facial movements. Scale, CC, 0.5; pupil size, 0.3 mm; facial movement, 1 a.u.

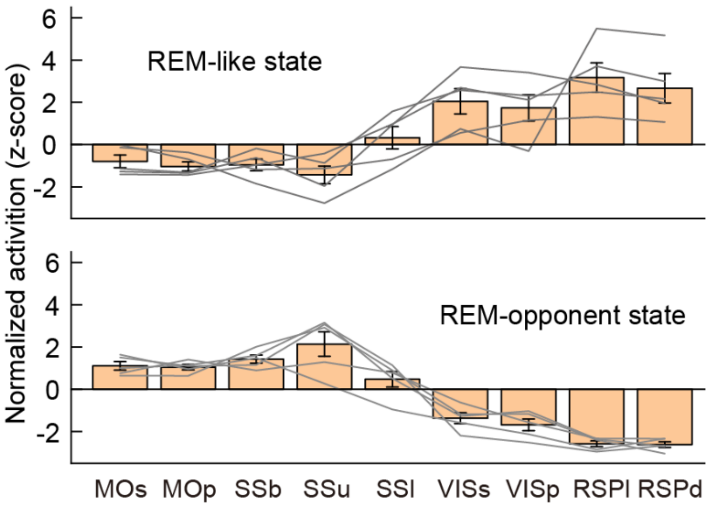

Supplementary Fig. 16 | Cortical activity during the REM-like and REM-opponent states (related to Fig. 5)

Shown is normalized cortical activity during the REM-like state and REM-opponent state. Each line represents data from individual image frames. Raw data are provided in a Source Data file. $n = 5$ REM-like or REM-opponent events, respectively. Data are mean ± SEM.

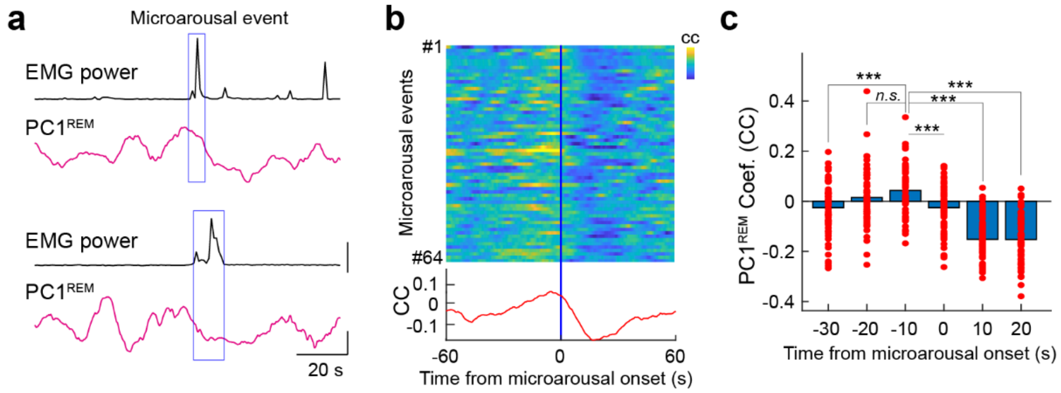

Supplementary Fig. 17 | PC1^{REM}-like activity and microarousal event (related to Fig. 5)

a Two examples showing PC1^{REM}-like activity during microarousal events (highlighted in the blue box). Sacle, EMG power 100 a.u.; PC1^{REM} coefficient (CC), 0.3. **b** PC1^{REM} CC aligned to the onset of microarousal events (n = 64 from 5 mice). Scale (CC), -0.4 – 0.4. **c** Quantification of change in PC1^{REM} from all five mice. Statistical comparison was performed between the 3rd group (from -10 s to onset of microarousal events) and the other five groups. ***$P < 0.001$; *n.s.*, $P > 0.05$ (one-way repeated measures ANOVA with Tukey's posthoc test). n = 64. Raw data for **c** are provided in a Source Data file.

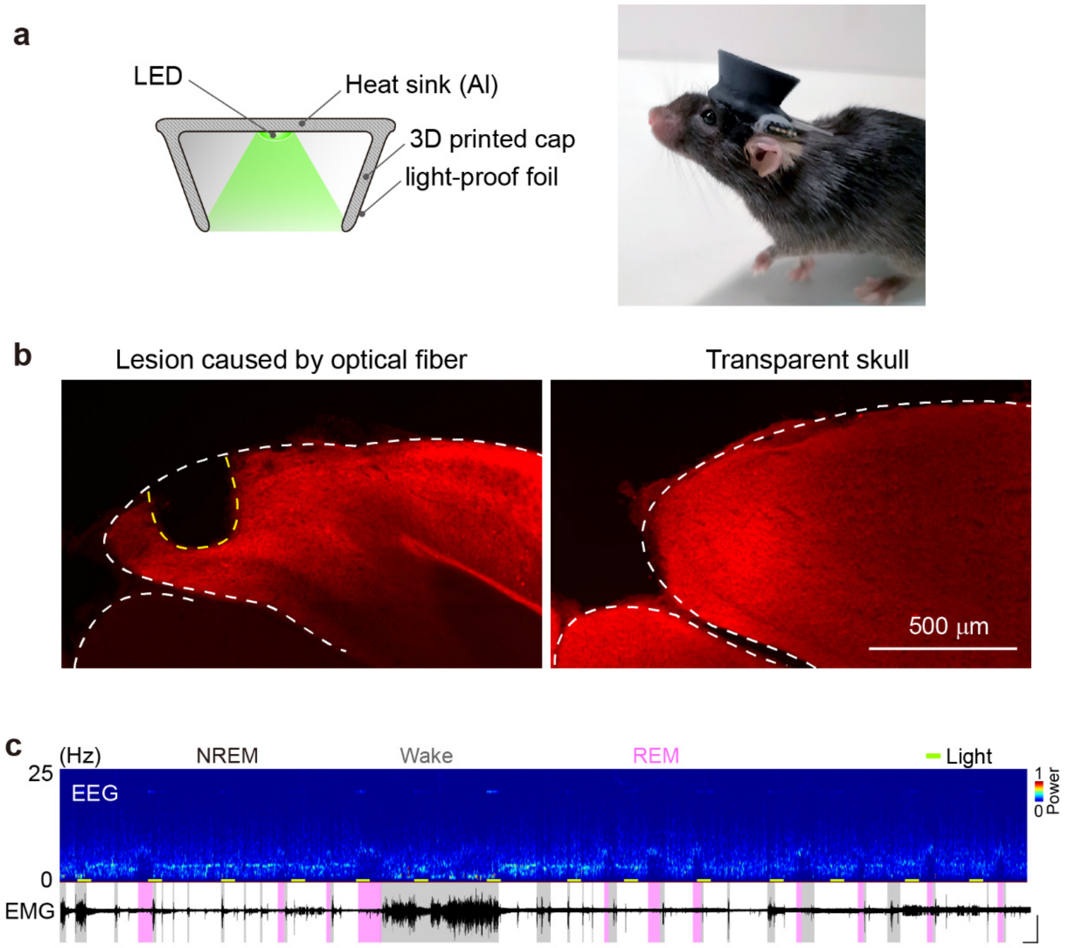

Supplementary Fig. 18 | Optogenetic inhibition of the occipital neocortex during the sleep-wake cycle (related to Fig. 7)

a Diagram of the light delivery device (left) and a photo of a mouse wearing the device right). **b** Fluorescent pictures of the RSP from experiments in which light was applied through an optical fiber (left) or a transparent skull (right). The considerable damage to the tissue was caused by fiber insertion, while no detectable damage occurred using the transparent skull method. 4 and 11 mice were used in the fiber insertion or transparent skull method, respectively. **c** An example showing optogenetic inhibition of the occipital neocortex from a mouse expressing ChrimsonR. Top to bottom, EEG power spectrogram, EMG (scale, 120 s, and 1 mV).

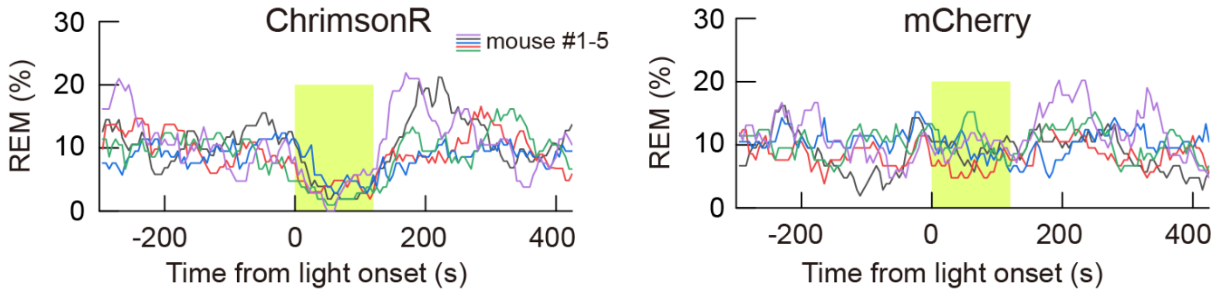

Supplementary Fig. 19 | Light-induced REM sleep modulation in individual mice (related to Fig. 7)

Shown is the modulation of REM sleep in each mouse during light stimulation (yellow shading) in mice expressing ChrimsonR (left) or mCherry (right). n = 25 sessions from 5 mice (5 sessions/mouse) for the ChrimsonR group; n = 24 sessions from 5 mice (4 or 5 sessions/mouse) for the mCherry group. Each line represents the average result from each mouse.

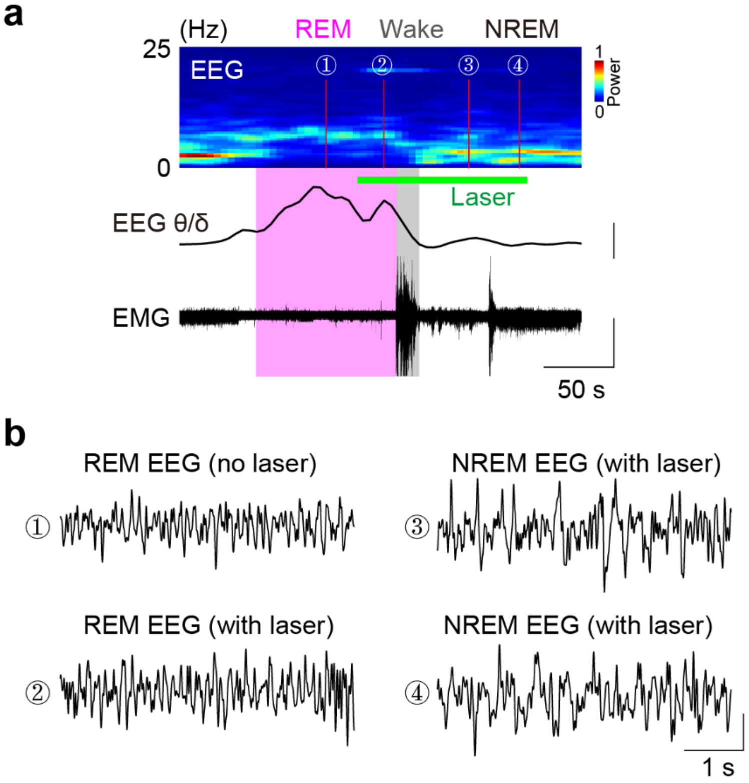

Supplementary Fig. 20 | Light stimulation does not affect NREM-REM classification (related to Fig. 7)

a An example showing EEG power spectrogram (up), EEG θ/δ ratio (middle; scale, 1), and EMG (low; scale, 0.5 mV). EEG traces from the four selected time points are shown in **b.** Scale in b, 50 μV

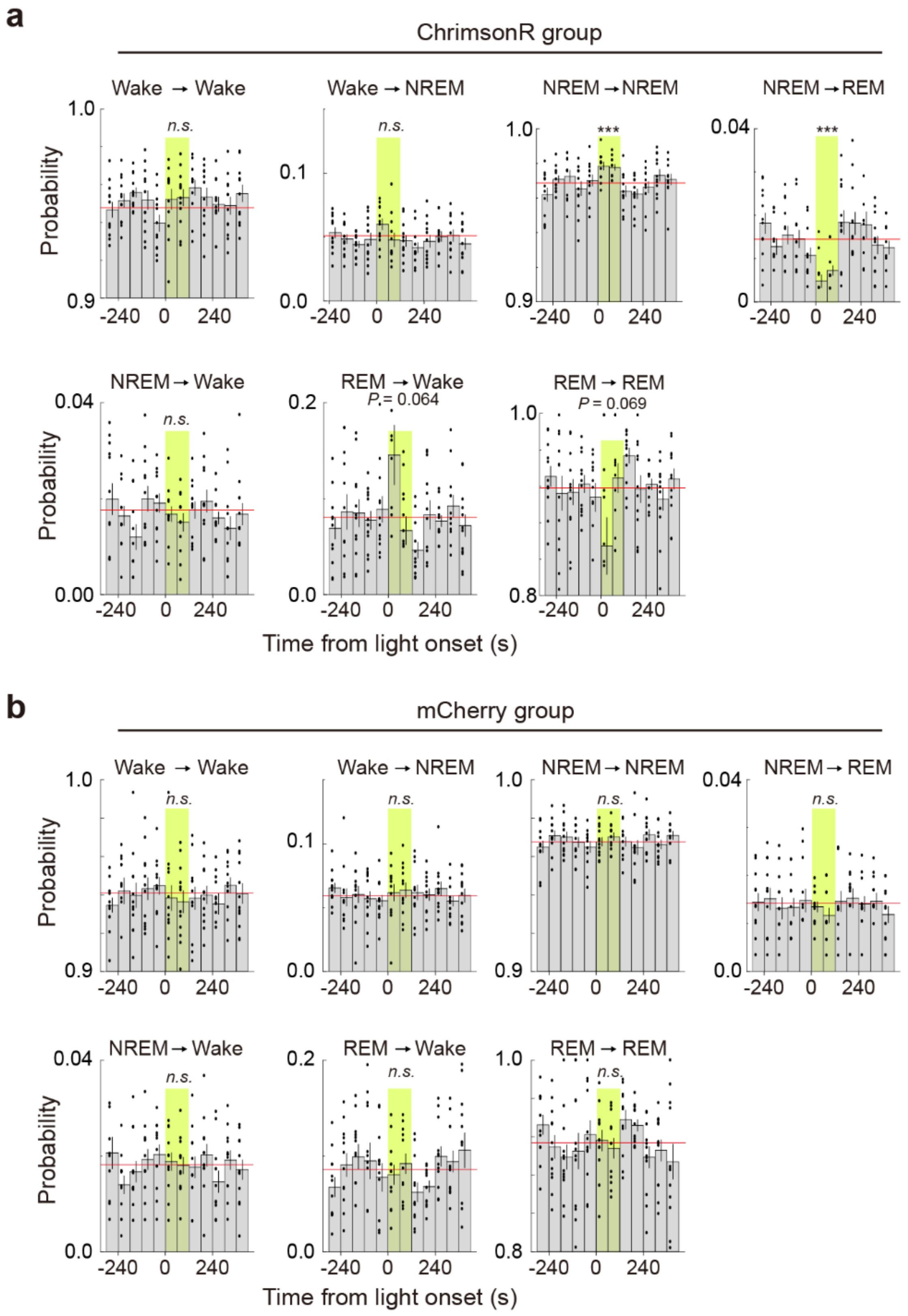

Supplementary Fig. 21 | Brain state transitions during optogenetic inhibition of the occipital activity (related to Fig. 7)

Shown is the probability of brain state transitions for the indicated pair of brain states. Red line, baseline transition probability. Yellow shading, light stimulation. Student's paired *t*-test or Wilcoxon signed-rank test. Data were from 25 sessions from 5 mice expressing ChrimsonR **(a)**, and 24 sessions from 5 mice expressing mCherry **(b)**. Part of the data for the ChrimsonR was already shown in Fig. 7, and these data were shown again to facilitate comparison with the mCherry group. Data are mean ± SEM. Raw data and exact *P* values are provided in a Source Data file.

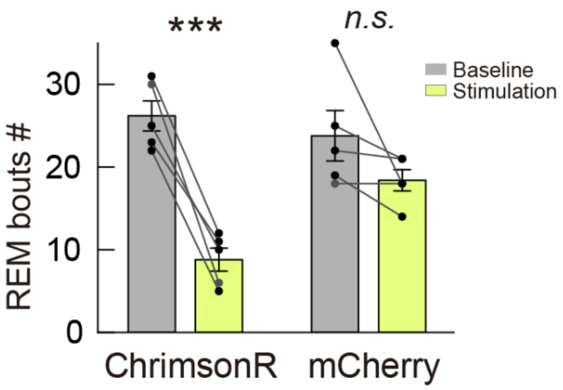

Supplementary Fig. 22 | Light stimulation reduced REM bout number (related to Fig. 7)

Shown is the mean number of REM bouts during stimulation and baseline in each experiment from ChrimsonR and mCherry groups. The selected baseline period (2-minutes after each light stimulation) had the same duration as the stimulation period. ChrimsonR: $P < 0.0001$; mCherry: $P = 0.15$; Paired t-test. Data were from 25 sessions from 5 mice expressing ChrimsonR and 24 sessions from 5 mice expressing mCherry. Data are mean ± SEM. Raw data are provided in a Source Data file.
