# Supplementary material for: REM sleep is associated with distinct global cortical dynamics and controlled by occipital cortex
Source: Nat Commun. 2022 Nov 12;13:6896. doi: 10.1038/s41467-022-34720-9 (PMC9653484; doi:10.1038/s41467-022-34720-9)
Supplement: Supplementary file 3 — Description of Additional Supplementary Files [file 41467_2022_34720_MOESM3_ESM.docx]

**Description of Additional Supplementary Files**

**Supplementary Movie 1:** Mesoscale Ca2+ imaging was performed through the transparent skulls of Thy1-GCaMP6s mice during the sleep-wake cycle. 470-nm light was used to excite GCaMP, and 405-nm light was used as a control. The image was acquired using a frame rate of 2.5 Hz and replayed ten times in real-time. The blood vessels were not masked in this movie.

**Supplementary Movie 2:** Highly structured cortical activity during the sleepwake cycle. The image was acquired using a frame rate of 10 Hz and replayed four times in real-time. The blood vessels were not masked in this movie.

**Supplementary Movie 3:** Cortical activity, eye movements, and EEG θ/δ ratio during REM sleep. The image was acquired using a frame rate of 10 Hz and replayed two times in real-time. The blood vessels were not masked in this movie.

**Supplementary Movie 4 and 5:** Cortical activity during REM sleep from two different mice. The movies show the spreading activation starting from the RSP. The image was acquired using a frame rate of 10 Hz and replayed in real-time. The blood vessels were not masked in this movie.
