# Supplementary material for: REM sleep is associated with distinct global cortical dynamics and controlled by occipital cortex
Source: Nat Commun. 2022 Nov 12;13:6896. doi: 10.1038/s41467-022-34720-9 (PMC9653484; doi:10.1038/s41467-022-34720-9)
Supplement: Supplementary file 9 — Reporting Summary [file 41467_2022_34720_MOESM9_ESM.pdf]

nature portfolio

Corresponding author(s): Min Xu
Siyu Zhang

Last updated by author(s): Oct 17, 2022

Reporting Summary

Nature Portfolio wishes to improve the reproducibility of the work that we publish. This form provides structure for consistency and transparency in reporting. For further information on Nature Portfolio policies, see our Editorial Policies and the Editorial Policy Checklist.

Statistics

For all statistical analyses, confirm that the following items are present in the figure legend, table legend, main text, or Methods section.

n/a	Confirmed	
☐	☒	The exact sample size (*n*) for each experimental group/condition, given as a discrete number and unit of measurement
☐	☒	A statement on whether measurements were taken from distinct samples or whether the same sample was measured repeatedly
☐	☒	The statistical test(s) used AND whether they are one- or two-sided<br>*Only common tests should be described solely by name; describe more complex techniques in the Methods section.*
☒	☐	A description of all covariates tested
☐	☒	A description of any assumptions or corrections, such as tests of normality and adjustment for multiple comparisons
☐	☒	A full description of the statistical parameters including central tendency (e.g. means) or other basic estimates (e.g. regression coefficient) AND variation (e.g. standard deviation) or associated estimates of uncertainty (e.g. confidence intervals)
☐	☒	For null hypothesis testing, the test statistic (e.g. *F*, *t*, *r*) with confidence intervals, effect sizes, degrees of freedom and *P* value noted<br>*Give P values as exact values whenever suitable.*
☒	☐	For Bayesian analysis, information on the choice of priors and Markov chain Monte Carlo settings
☐	☒	For hierarchical and complex designs, identification of the appropriate level for tests and full reporting of outcomes
☐	☒	Estimates of effect sizes (e.g. Cohen's *d*, Pearson's *r*), indicating how they were calculated

Our web collection on statistics for biologists contains articles on many of the points above.

Software and code

Policy information about availability of computer code

Data collection	EEG/EMG data and optogenetic stimulation data were collected using 'OpenEX' (Tucker-Davis Technologies, ver: 2.31) and RZ5 system (Tucker-Davis Technologies).<br>EEG/EMG data during wildfield imaging were collected using RHD acquisition board and RHX software (Intan Technologies, ver: 3.03).<br>The calcium imaging data were collected using Micro-Manager (NIH, ver: 1.4).<br>The histology data were collected using VS120 (Olympus).
Data analysis	Data analysis were performed using custom scripts in MATLAB 2019b.<br>The atlas registration was performed using open source software package 'ANTS' (http://stnava.github.io/ANTs/)<br>The eye movement data was extracted using open source software package 'FaceMap' (https://github.com/MouseLand/facemap)<br>Figures were prepared using 'OriginLab' (OriginLab Corp., 2019b), 'MATLAB', and 'Illustrator' (Adobe, CS6).

For manuscripts utilizing custom algorithms or software that are central to the research but not yet described in published literature, software must be made available to editors and reviewers. We strongly encourage code deposition in a community repository (e.g. GitHub). See the Nature Portfolio guidelines for submitting code & software for further information.

Data

Policy information about availability of data

All manuscripts must include a data availability statement. This statement should provide the following information, where applicable:

- Accession codes, unique identifiers, or web links for publicly available datasets
- A description of any restrictions on data availability
- For clinical datasets or third party data, please ensure that the statement adheres to our policy

Statistical source data for all figures is available for download via links associated with this paper on the Nature Commications website. All raw data are available from the corresponding authors upon reasonable request.

Human research participants

Policy information about studies involving human research participants and Sex and Gender in Research.

Reporting on sex and gender	NA
Population characteristics	NA
Recruitment	NA
Ethics oversight	NA

Note that full information on the approval of the study protocol must also be provided in the manuscript.

Field-specific reporting

Please select the one below that is the best fit for your research. If you are not sure, read the appropriate sections before making your selection.

[x] Life sciences [ ] Behavioural & social sciences [ ] Ecological, evolutionary & environmental sciences

For a reference copy of the document with all sections, see nature.com/documents/nr-reporting-summary-flat.pdf

Life sciences study design

All studies must disclose on these points even when the disclosure is negative.

Sample size	We did not perform a calculation on the sample size. We used a sample size comparable to previous studies using similar techniques and animal models (Xu et al., Nature Neuroscience, 2015; Pedersen et al., Nature Communications, 2017; Gent et al., Nature Neuroscience, 2018)
Data exclusions	No data was excluded from this study.
Replication	All the key experiments in the current study contains >= 3 animals, which were tested in >= 2 independent repeats of experiments. The exact number of mice/recording sessions/cells in a specific experiment was reported in the text and/or figure legends.
Randomization	Animals in mesoscale calcium imaging were not allocated into different groups. Animals in optogenetic manipulation experiments were randomly assigned to a virus cohort (e.g. ChrimsonR versus mCherry).
Blinding	In the studies, investigators were blinded to groups for data collection and analysis.

Reporting for specific materials, systems and methods

We require information from authors about some types of materials, experimental systems and methods used in many studies. Here, indicate whether each material, system or method listed is relevant to your study. If you are not sure if a list item applies to your research, read the appropriate section before selecting a response.

Materials & experimental systems

n/a	Involved in the study
☒	☐ Antibodies
☒	☐ Eukaryotic cell lines
☒	☐ Palaeontology and archaeology
☐	☒ Animals and other organisms
☒	☐ Clinical data
☒	☐ Dual use research of concern

Methods

n/a	Involved in the study
☒	☐ ChIP-seq
☒	☐ Flow cytometry
☒	☐ MRI-based neuroimaging

Animals and other research organisms

Policy information about studies involving animals; ARRIVE guidelines recommended for reporting animal research, and Sex and Gender in Research

Laboratory animals	Details of the mice used in the current study (the age reported here is at the time of data collection):<br>Fig.1-6 (except Fig. 5g-j) and Supplementary Fig. 1-17: 4 Thy1-GCaMP6s-het mice, Age(weeks)(number of mice: male / female): 18 (2/0), 8(2/0); 1 Thy1-GCaMP6s-homo mice, 24(1/0). Same format was used to report age of the mice in all our experiments.<br>Fig. 7a-f and Supplementary Fig. 18-22: 5 GAD2-Cre mice for ChrimsonR group, 12(4/0), 9(0/1); 5 GAD2-Cre mice for mCherry group, 14(0/2), 8(2/1)<br>Fig. 4g-i: 3 Thy1-GCaMP6s-het mice, 20(2/0), 10(1/0)<br>Fig. 7g-i: 6 C57BL/6 (WT) mice, 12(6/0)<br>Fig. 7j-l: 6 GAD2-Cre mice, 29(0/6)
Wild animals	No wild animals were used in this study.
Reporting on sex	Both male and female mice were used.
Field-collected samples	No field-collected samples were used in this study.
Ethics oversight	All experimental procedures followed the National Institutes of Health guidelines and were approved by the Animal Care and Use Committee at the Institute of Neuroscience, Chinese Academy of Sciences.

Note that full information on the approval of the study protocol must also be provided in the manuscript.
